# Convective Drying of Purple Basil (*Ocimum basilicum* L.) Leaves and Stability of Chlorophyll and Phenolic Compounds during the Process

**DOI:** 10.3390/plants12010127

**Published:** 2022-12-27

**Authors:** Rosane Patricia Ferreira Chaves, Adriano Lucena de Araújo, Alessandra Santos Lopes, Rosinelson da Silva Pena

**Affiliations:** 1 Graduated Program in Food Science and Technology, Institute of Technology, Federal University of Pará (UFPA), Belém 66075-110, PA, Brazil; 2 Faculty of Food Engineering, Institute of Technology, Federal University of Pará (UFPA), Belém 66075-110, PA, Brazil

**Keywords:** drying kinetics, modeling, moisture sorption, biocompounds, thermodegradation

## Abstract

This study evaluated the effect of convective drying on the degradation of color and phenolic compounds of purple basil (*Ocimum basilicum* L.) leaves, and the hygroscopic behavior of dried leaves. The fresh leaves underwent drying at 40 °C, 50 °C, 60 °C, and 70 °C. Degradation of chlorophyll, flavonoids, and phenolic compounds were evaluated during drying and the hygroscopicity was evaluated through the moisture sorption isotherms. The drying mathematical modeling and the moisture sorption data were performed. The effective diffusivity for the drying increased from 4.93 × 10^−10^ m^2^/s at 40 °C to 18.96 × 10^−10^ m^2^/s at 70 °C, and the activation energy value (39.30 kJ/mol) showed that the leaves present temperature sensibility. The leaves dried at 40 °C had less degradation of phenolic compounds and color variation, but the drying process was too slow for practical purposes. Modified Page, Diffusion Approximation, and Verna models had excellent accuracy in drying kinetics. The isotherms showed that, in environments with relative humidity above 50%, the purple basil leaves are more susceptible to water gain, and at 8.83 g H_2_O/100 g db moisture, it guarantees the microbiological stability of the dried leaves. The Oswin model was the most suitable for estimating the moisture sorption isotherms of the dried leaves.

## 1. Introduction

The purple basil (*Ocimum basilicum* L.), also called ‘alfavaca-roxa’ and ‘alfavaca-comum’, is a plant species adapted to the Eastern Amazon, used in traditional medicine as a stimulant, in the treatment of hypertension and renal failure, against coughs, and in the form of baths to combat colds [1,2,3].

The purple basil presents in its composition fibers, carbohydrates, proteins, and minerals such as iron, calcium, and phosphorus [2]. Phenolic compounds are the secondary metabolites in this plant species, especially phenolic acids, flavonoids, tannins, and polyphenols, which act as reducing agents, metal chelators, and free radical scavengers [4]. The presence of these compounds gives Ocimum leaves antimicrobial, antioxidant, anti-inflammatory, and antihyperglycemic potential [2,5,6].

Due to medicinal properties, functional claims, and aromatic characteristics, basil has been gaining economic prominence due to the numerous possibilities of use in the pharmaceutical and food industries. Although basil occurs throughout the year, post-harvest treatments are necessary to control microbiological and biochemical changes and prolong its shelf life. Drying is a preservation method frequently used for the dehydration of leaves, and the literature shows that it has been used as a pre-treatment of basil leaves, to obtain essential oil [7] and for the infusion preparation [8].

Drying is a unit operation that removes water from cells and tissues. In this process, the transfer of heat and mass occurs simultaneously between the product and the air-drying. The increase in air-drying temperature causes the movement of water molecules and an increase in the partial pressure of water vapor in the product, which promotes a reduction in the water content and water activity (a_w_) [9]. The free water content is reduced to levels that can ensure a decrease in microbiological activity and enzymatic degradation, contributing to an increase in the shelf life of the product, in addition to favorably minimizing the packaging and transportation of the product, due to the reduction in volume [10,11].

On the other hand, the drying process can cause physical, chemical, and sensorial changes in the product; therefore, minimizing such effects is important to ensure the quality of the final product [10]. The degradation of compounds sensitive to heat, light, and oxygen, such as phenolic compounds, vitamins, and pigments, can be reduced or prevented by controlling the temperature, time, and drying method applied [12].

In the drying of aromatic plants, mathematical modeling predicts the drying behavior and can help in the definition of conditions for the process, capable of guaranteeing the preservation of the characteristics of the product, as well as a high efficiency for the drying [11,13]. Semi-theoretical models are the most used to describe the drying phenomenon. These models are based on the theory and data of drying kinetics, and they are derived from the simplification of Fick’s second law of diffusion or from the modification of simplified models. Such models consider that at a decreasing drying rate, the movement of liquid or vapor occurs in a porous solid in a given period, which is represented by the change in relative humidity [14].

To preserve the characteristics of an aromatic plant after drying, it is important to study the hygroscopic behavior of the product, through the moisture sorption isotherms. These isotherms define the relationship between the moisture content and the water activity (a_w_) of the product, and make it possible to determine the moisture content at which the product presents the greatest stability to degradative processes. Additionally, moisture sorption isotherms allow for the definition of drying conditions, as well as the packaging and storage, for the product [15,16].

There are several scientific works that apply different drying methods to the genus Ocimum and evaluate the post-drying behavior. Seczyk, Ozdemir, and Kolodziej [17] analyzed the effect of convective drying at 40 °C for 48 h, freezing at −20 °C, and lyophilization over the in vitro bioaccessibility of phytochemicals in basil, and found that convective drying achieved greater bioaccessibility of phytochemicals compared with those processed at low temperatures, despite having the highest amount of phenolics and antioxidant activity. Diaz-Maroto et al. [18] studied changes in basil aromas after convective drying at 45 °C, air-drying at room temperature, and lyophilization, and demonstrated that the temperature caused a decrease in volatile compounds and changes to the odor of the leaves.

Few works with Ocimum present data with moisture sorption isotherms. Lima-Corrêa et al. [19] evaluated the influence of drying basil leaves with fluidized beds and vibrofluidized on the volatile oil composition and color change, and applied the moisture sorption isotherm to evaluate the equilibrium moisture content as a function of temperature and relative humidity. Pääkkönen, Malmsten, and Hyvönen [20], in their work on basil and marjoram, concluded that the drying conditions, such as air-drying and lyophilization, modified the water adsorption in basil, and that hermetically closed packages can maintain the intensity of odor and flavor.

Based on the above, the objective of this work was to study the effect of convective drying, over a wider temperature range (40–70 °C), on the degradation of chlorophyll and phenolic compounds of the purple basil (*O. basilicum*), as well as to study the hygroscopic behavior to define the drying and storage conditions for the product. For the first time, this study presents practical data for the convective drying of purple basil leaves, as well as practical conditions for the storage of the dried leaves.

## 2. Results and Discussion

### 2.1. Characterization of Purple Basil Leaves

The results of the proximate composition and physical-chemical properties of the purple basil leaf are shown in Table 1. The average moisture content (83.56%) of the purple basil (*O. basilicum*) leaves was at the same order of magnitude as the value observed for basil (*O. basilicum*) by Reis et al. [21] (82.7%). On the other hand, Roberto et al. [22] observed higher values of moisture content (94.12%) for *O. basilicum*. Other plant species, typical from the Amazon region, had high moisture contents, such as jambu (*Acmella oleracea*) (92.99%) [23] and chicory (*Eryngium foetidum*) (87%) [24].

The average contents of ash (1.63%), total proteins (0.57%), and total lipids (0.15%) observed in purple basil leaves were lower than the values reported by Oluwole et al. [25] for *Ocimum gratissimum* L. (7.12%, 3.71%, and 3.59%, respectively). The total carbohydrates in purple basil (3.14%) were also lower than that reported for *O. basilicum* leaves (5.03%) by Siti Mahirah et al. [26].

Total fibers make up approximately 11% of the composition of purple basil leaves. This content was similar to that observed in the leaves of *O. gratissimum* L. (10.34%) by Almeida et al. [27] and higher than that reported (7.80%) by Borges et al. [24]. The total energetic value of the purple basil leaves was 16.11 kcal/100 g, which was lower than that found by Borges et al. [28] (278.34 kcal/100 g) for *O. gratissimum* leaves.

The purple basil leaf had a 6.75 pH, an acidity of 0.72 mEq NaOH/100 g, and a total soluble solids content of 5.20 °Brix. According to Chitarra and Chitarra [29], organic acids are dissolved in cellular vacuoles that give the plant tissue acidity and cause the plant’s pH tissue to vary from 5 to 7. Henrique, Ferreira, and Nunes [30] observed an average pH value of 6.43, an acidity of 0.195 mEq NaOH/100 g, and soluble solids of 3.0 °Brix, for organic basil leaves (*O. basilicum* L.). The high value of a_w_ (0.99) indicates that the purple basil leaves are very susceptible to the action of microorganisms, enzymes, and degradative processes in general [31]. Thus, drying is a process capable of reducing a_w_, at levels that ensures the degradative stability and thus increases the useful life of this leaf.

The different values observed for the composition and physical–chemical properties of the purple basil leaves when compared with the leaves of other plant species in the same genus and with plant species of other genera can be attributed to the place of cultivation, the soil and climate conditions, and the species or genus [32,33].

### 2.2. Drying of Purple Basil Leaves

#### 2.2.1. Drying Kinetics

**Figure 1:**
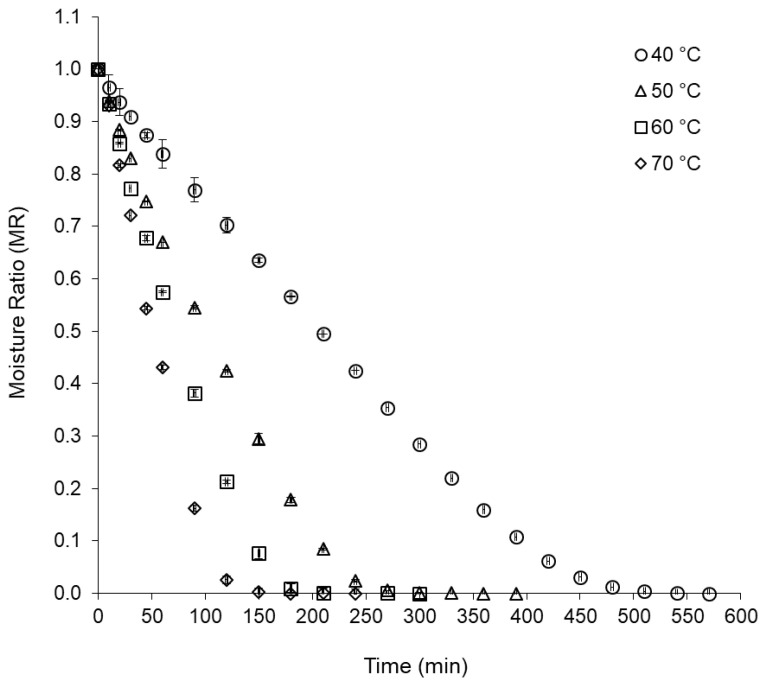
Drying curves of purple basil leaves at temperatures of 40 °C, 50 °C, 60 °C, and 70 °C.

Figure 1 shows the drying curves of the basil leaves at temperatures of 40 °C, 50 °C, 60 °C, and 70 °C. In the initial stage of drying, a linear behavior was observed between moisture ratio (MR) and time, indicating that the process occurred at a constant drying rate, which increased with increasing temperature (increase in the straight slope) and it was much lower for drying at 40 °C, when compared with drying at temperatures from 50 °C to 70 °C. In the next drying stage, an exponential behavior of MR occurred with time, indicating that the process occurred with decreasing drying rates, for all conditions studied.

Similar drying behaviors were observed for rosemary leaves (*Rosmarinus officinalis* L.) dried by hybrid convective and microwave drying processes, under a vacuum [34]; for sage leaves (*Salvia officinalis*) dried in a cabin dryer [35]; and for basil leaves (*O. basilicum* L.) dried by microwave [36]. Mbegbu, Nwajunka, and Amaefule [37] reported that diffusion was the predominant mechanism for moisture transport during the thin layer drying of *O. gratissimum* and *Ocimum africanum* leaves.

Concerning the total drying time, a significant reduction was observed with the increase in the drying temperature, requiring a drying time of 2 h and 30 min at 70 °C; 3 h and 30 min at 60 °C; 4 h and 30 min at 50 °C; and 8 h and 30 min at 40 °C. Thus, there was a 40% increase in drying time at 60 °C compared with 70 °C, 29% at 50 °C compared with 60 °C, and 89% at 40 °C compared with 50 °C. These results indicate that the convective drying of purple basil leaves at 40 °C is a slow process, not suitable for practical purposes, except when higher temperatures degrade chemical compounds of interest. According to Babu et al. [31], air temperature and drying time can cause physical and structural changes and chemical losses due to the evaporation of volatile compounds, as they affect quality attributes, flavor, and the nutritional value of leaves. In this context, Rocha, Melo, and Radünz [38] reported that at 50 °C, the losses of chemical constituents were minimal during the drying of medicinal plants.

#### 2.2.2. Effective Diffusivity and Activation Energy

Effective diffusivity (*D_eff_*) of water, for drying purple basil leaves, was 4.93 × 10^−10^ m^2^/s at 40 °C, 9.94 × 10^−10^ m^2^/s at 50 °C, 13.87 × 10^−10^ m^2^/s at 60 °C, and 18.96 × 10^−10^ m^2^/s at 70 °C. The behavior observed for *D_eff_* indicated that the increase in drying temperature favored the elimination of water from the leaves, confirming the behavior of the drying curves (Figure 1). According to Fernandez et al. [39], the *D_eff_* can vary depending on the internal conditions and the product structure, with the moisture content of the material, and with the drying temperature.

Seyedabadi [40] studied the effect of microwave power on the drying of basil leaves (*O. basilicum* L.) and observed that increasing power (from 90 W to 900 W) caused an increase in *D_eff_* (from 1.62 × 10^−10^ m^2^/s to 7.65 × 10^−10^ m^2^/s). Mbegbu, Nwajunka, and Amaefule [37] observed *D_eff_* values between 4.76 × 10^−13^ and 1.47 × 10^−12^ m^2^/s for the drying of sweet basil leaves (*O. gratissimum*), and between 4.83 × 10^−13^ and 2.06 × 10^−12^ m^2^/s for lemon basil leaves (*O. africanum*), both carried out in a vacuum oven, with temperatures between 30 °C and 70 °C. These *D_eff_* values were lower than the values observed in the drying of purple basil leaves, while the *D_eff_* values observed by Seyedabadi [40] were of the same magnitude order.

The activation energy (*E_a_*), estimated for the drying process of purple basil leaves, was 39.30 kJ/mol. This value was higher than those reported for the drying of scent leaves (*O. gratissimum*) (*E_a_* = 25.01 kJ/mol) and lemon basil leaves (*O. africanum*) (*E_a_* = 32.35 kJ/mol) [37]. The highest *E_a_* value indicates that the purple basil leaves are more sensitive to the drying air temperature.

#### 2.2.3. Modeling of Drying Curves

**Figure 2:**
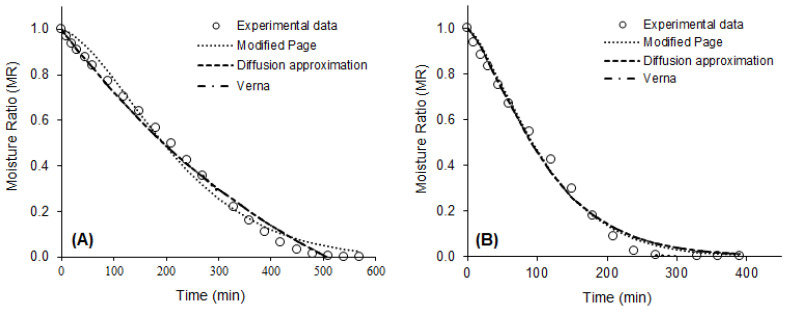

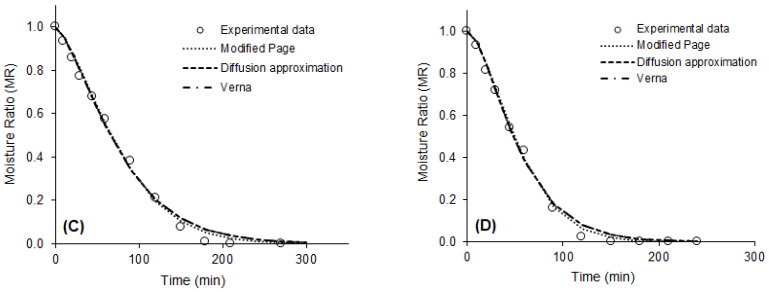
Experimental drying data and drying curves predicted by the modified Page, Diffusion Approximation, and Verna models. (**A**) 40 °C; (**B**) 50 °C; (**C**) 60 °C; (**D**) 70 °C.

The values of the statistics used to evaluate the quality of the mathematical fits and the parameters of the eight models fitted to the drying data of the purple basil leaves are presented in Table 2. Values of R^2^ > 0.95, χ^2^ < 0.007, and RMSE < 0.08 indicated that all models tested were capable of predicting, with good accuracy, the drying curves of purple basil leaves, in all conditions studied. However, the best fits for all drying conditions were observed for the modified Page models, with two parameters (R^2^ > 0.99, χ^2^ < 0.0014 and RMSE < 0.04), Diffusion Approximation (R^2^ > 0.99, χ^2^ < 0.0013, RMSE < 0.03), and Verna (R^2^ > 0.99, χ^2^ < 0.0013 and RMSE = 0.03), with three parameters. These models estimated with excellent precision the drying kinetics of the product, at all temperatures evaluated, as can be seen in Figure 2.

Altay, Hayaloglu, and Dirim [36] studied the drying of basil leaves (*O. basilicum* L.) by different methods. These authors observed that the Henderson and Pabis were the models that best described drying that occurred from the sun, the Logarithmic model presented the best fit for the freeze-drying data, and the modified Page was the model that more accurately predicted the drying in a convective dryer and in a microwave oven. Alibas et al. [41] observed that the modified Page model accurately described the natural drying curve of basil leaves.

### 2.3. Stability of Chlorophyll and Phenolic Compounds

**Figure 3:**
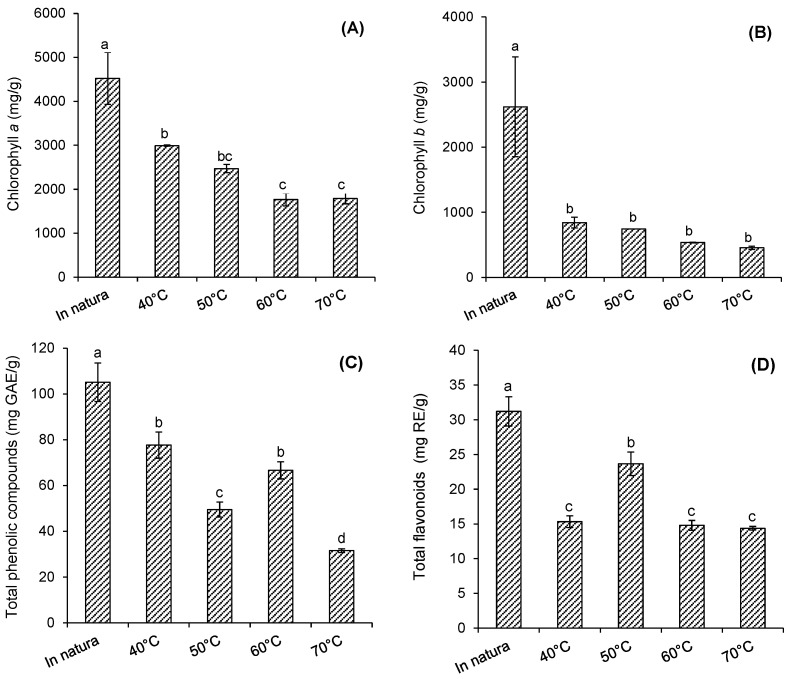
Behavior of chlorophyll, phenolic compounds, and flavonoids in fresh purple basil leaves and those dried at 40 °C, 50 °C, 60 °C, and 70 °C. (**A**) chlorophyll *a* (mg/g), (**B**) chlorophyll *b* (mg/g), (**C**) total phenolic compounds (mg GAE/g), and (**D**) total flavonoids (mg/g). Means with the same letters in the same graph do not differ statistically (*p* ≤ 0.05) for Tukey’s test.

Figure 3 shows the levels of chlorophyll *a*, chlorophyll *b*, total flavonoids (TF), and total phenolic compounds (TPC) in fresh purple basil leaves dried at 40 °C, 50 °C, 60 °C, and 70 °C. The content of chlorophyll *a* (4522.94 mg/g) (Figure 3A) in fresh basil leaves was higher than the content of chlorophyll *b* (2619.39 mg/g) (Figure 3B), and the value of TPC (105.1 mg GAE/g) (Figure 3C) was also higher than the total flavonoid content (TF) (31.19 mg RE/g) (Figure 3D).

The chlorophyll *a* content of fresh purple basil leaves was significantly reduced (*p* ≤ 0.05) when the leaves were dried under different temperature conditions (Figure 3A). For the leaves dried at 40 °C, a degradation of 33.8% in chlorophyll *a* was observed, in relation to fresh leaves. When the drying temperature increased to 50 °C, a degradation of 45.4% was observed, and for dryings carried out at 60 °C and 70 °C, the observed losses corresponded to 60%, approximately (Figure 3A). For chlorophyll *b*, there was a degradation of 68% in the drying at 40 °C, in relation to fresh leaves (*p* ≤ 0.05), and no significant differences were observed among chlorophyll *b* values, for the leaves dried at the different temperatures (*p* > 0.05) (Figure 3B).

Chlorophylls are natural pigments responsible for the green color of leaves. When exposed to the action of temperatures, such as those practiced in drying processes, they can undergo degradation due to conversion into pheophytin, which has a green-brown color, or can release substrates for enzymatic browning reactions [42]. Alibas et al. [41] analyzed the chlorophyll degradation of *O. basilicum* L. leaves during natural, convective, and microwave drying. The authors observed that the chlorophyll values were significantly reduced by all drying methods.

As observed for chlorophyll, all drying conditions promoted a significant reduction (*p* ≤ 0.05) in the TPC (26.1% at 40 °C) (Figure 3C) and TF (50.9% at 40 °C) (Figure 3D). The increase in temperature, in turn, caused a reduction in the TPC (*p* ≤ 0.05) with a degradation of 70%, at 70 °C. On the other hand, there was no tendency to decrease the FT (*p* > 0.05) at the temperature range used.

Phenolic compounds and flavonoids are among the main chemical compounds found in basil leaves [4,43]. Sharma, Bhatia, and Kaur [44] reported the degradation of phenolic compounds and flavonoids during the drying of basil leaves by microwave, by exposure to the sun, and in a tray dryer, at temperatures of 45 °C, 50 °C, and 55 °C. They observed that the highest level of degradation occurred in tray drying at 55 °C. Other authors have also reported the loss of phenolic compounds and flavonoids during thermal processes [45,46].

Therefore, it was observed that convective drying promoted a reduction in the contents of chlorophylls, TPC, and TF, when compared with fresh purple basil leaves; however, drying is a necessary operation for the post-harvest preservation of leaves, as well as a pre-treatment for other processes. Research carried out with medicinal and aromatic plants indicated that temperatures of 40 °C and 50 °C decreased the loss of chemical compounds and nutritional quality [38,47]. Thus, according to the results, drying purple basil leaves at 40 °C for 8 h and 30 min can be applied for less degradation of chlorophylls and TPC.

### 2.4. Color Measurements

The instrumental color parameters a*, b*, C*, L*, *h°*, and ΔE values, for fresh and dried purple basil leaves at 40 °C, 50 °C, 60 °C, and 70 °C, are presented in Table 3. The color pattern indicated that the fresh basil leaves have a light green color. However, the values of all the color parameters changed significantly (*p* ≤ 0.05) after the leaves were submitted to drying, even for drying at 40 °C. There was an increase in the value of a* and decreases in the values of the other parameters (b*, C*, L*, and *h°*). Regarding the drying temperature, there was no effect on a* (*p* > 0.05), but there was a tendency to decrease in the values of b* and L* (*p* ≤ 0.05), with increasing temperatures. Consequently, a tendency of decrease in the value of C* was also observed with the increase in the temperature; however, there was no significant variation in the parameter *h°*, in the range of drying temperatures studied. The values of ΔE, in turn, indicated that the increase in the drying temperature promoted a degradation in the color of the leaves. According to the color parameters, the purple basil leaves lost their greenish color characteristic (*h°* = 123.3) and adopted a light yellow color after drying (average value of *h°* = 88.8).

### 2.5. Hygroscopic Behavior of Purple Basil Leaves

#### 2.5.1. Moisture Sorption Isotherms

**Figure 4:**
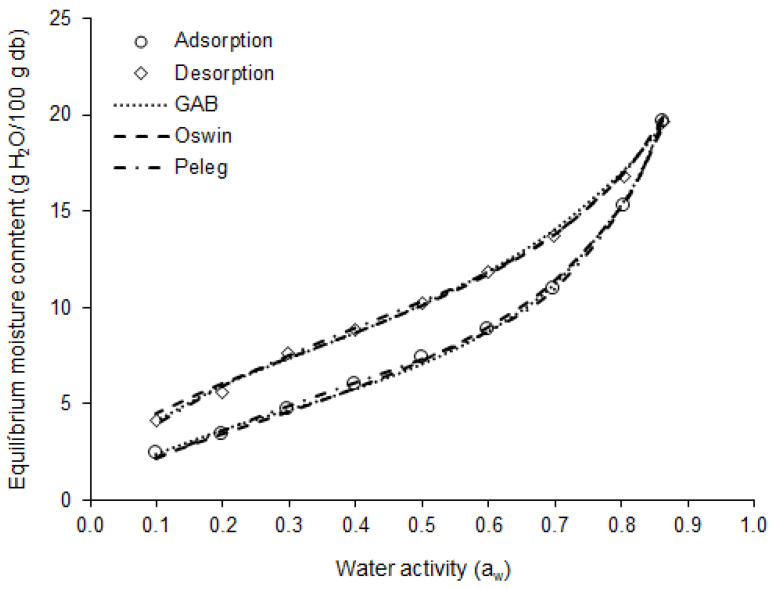
Moisture sorption isotherm at 25 °C for purple basil leaves: experimental data and curves obtained by the GAB, Oswin, and Peleg models.

The moisture adsorption and desorption isotherms of the basil leaves at 25 °C are shown in Figure 4. In the adsorption isotherm, the equilibrium moisture content (m) increased linearly with a_w_, between 0.1 and 0.5 a_w_ (Δm = 5 g H_2_O/100 g db), when the behavior became exponential, up to 0.9 a_w_ (Δm = 12.3 g H_2_O/100 g db). This behavior indicates that dried basil leaves will be more susceptible to moisture gain when exposed to an environment with relative humidity (RH) above 50%.

The moisture adsorption and desorption isotherms showed type II behavior (sigmoid shape), which is typical of biological materials [48]. Type II isotherms were observed by Lima-Corrêa et al. [19] for basil leaves (*O. basilicum*); by Canabarro et al. [49] for pitanga leaves (*Eugenia uniflora* L.); by Santos et al. [50] for jambu leaves (*Acmella oleracea*); and by Martins et al. [51] for guaco leaves (*Mikania glomerata* Sprengel).

The monolayer moisture content (m_o_) for adsorption indicated that 4.27 g H_2_O/100 g db is the moisture content level at which the dried purple basil leaves present the greatest stability under physical, chemical, and biological changes [52]. However, the adsorption isotherm indicates that the dried leaves will be microbiologically stable when they present a moisture content of less than 8.83 g H_2_O/100 g db (a_w_ < 0.6), if stored at 25 °C [53]. In turn, the value of m_o_ for desorption indicated that, when the purple basil leaves are subjected to drying, the moisture of the product should not reach moisture levels lower than 5.96 g H_2_O/100 g db, to avoid unnecessary power consumption, since below m_o_ the water is strongly bound to the product.

#### 2.5.2. Modeling of Moisture Sorption Isotherms

The results of the mathematical models’ fits to the experimental moisture adsorption and desorption data for dried purple basil leaves are presented in Table 4. The statistics used to assess the quality of the models’ fit indicated that the Oswin (R^2^ > 0.99, P < 3.1, RMSE < 0.25), GAB (R^2^ > 0.99, P < 2.4, RMSE < 0.23), and Peleg (R^2^ > 0.99, P < 2.7, RMSE < 0.15) models were able to describe with excellent accuracy the moisture adsorption and desorption isotherms of the dried basil leaves, as can be seen in Figure 4. The GAB and Peleg equations are more difficult to solve mathematically, as they are models with three and four parameters, respectively. In turn, as the Oswin is a model with two parameters, it is easy to solve it mathematically. Thus, for practical purposes, the Oswin model is the most suitable for estimating the moisture adsorption and desorption isotherms of the dried purple basil leaves.

Bensebia and Allia [54] observed that the GAB model was the one that best described the moisture adsorption and desorption isotherms of rosemary leaves, for a temperature range from 30 °C to 50 °C. Argyropoulos et al. [55] evaluated the fits of five moisture sorption models and observed that the modified Oswin model was the one that best fitted the moisture sorption data of lemon balm leaves, in the temperature range from 25 °C to 45 °C. Martins et al. [51], in turn, reported that the Oswin model was the one that best described the moisture sorption isotherms of guaco leaves, for a temperature range from 40 °C to 70 °C.

## 3. Materials and Methods

### 3.1. Plant Material

Purple basil (*Ocimum basilicum* L.) was collected in the municipality of Santa Izabel do Pará (1°23′00.0″ S 48°05′49.0″ W), located in the state of Pará, Brazil. Five plants were selected per production row, in a total of four rows. During the collection, part of the plant material was used for identification, exsiccate preparation, and deposit in the Marlene Freitas da Silva (MFS) herbarium of the State University of Pará—UEPA (Register MFS009423). Another part of the sample was immediately packed in a package that allowed air circulation and transported to the Federal University of Pará—UFPA (1°28′33.1″ S 48°27′26.2″ W). The samples (leaves with branches) were washed with running water to eliminate surface dirt. The leaves were then separated from the branches, and the torn, dark, and yellowed leaves were discarded. The selected leaves were submitted for sanitization in sodium hypochlorite solution with 100 mg/L of active chlorine for 15 min, followed by washing to eliminate residual chlorine. Part of these leaves was used for characterization, and another part was used for the drying experiments. The access to the species is registered in the National System for the Management of Genetic Heritage and Associated Traditional Knowledge—SISGEN, under the following registration: A8627A9.

### 3.2. Characterization of the Leaves

#### 3.2.1. Chemical Composition and Physical–Chemical Analysis

The fresh purple basil leaves were submitted for analysis of moisture content (method n° 920.151), ash (method n° 940.26), total proteins (method n° 920.152) (nitrogen-protein conversion factor of 5.75), pH (method n° 981.12), total titratable acidity (method n° 942.16), and total soluble solids (method no. 932.12), according to AOAC [56]. Total lipids were determined by the Bligh–Dyer method [57], total carbohydrates were estimated by difference, and total energy value was calculated according to the general Atwater conversion factors [58]. The water activity (a_w_) was determined by direct reading in a digital thermohygrometer (Decagon, Aqualab 4TEV, Pullman, EUA) at 25 °C.

#### 3.2.2. Determination of Chlorophyll a and Chlorophyll b

The quantification of chlorophyll *a* and chlorophyll *b* was performed using the method proposed by Lichtenthaler and Buchmam [59]. The extraction of these pigments was carried out with 95% ethanol, followed by centrifugation (KASVI, K14-0815P). The reading of the supernatant was performed in a spectrophotometer (BEL, Photonics, BEL Engineering, Monza, Italy), at wavelengths of 652.2 nm and 665.2 nm. The equations 1 and 2 were used to calculate the two fractions.

(1) $$C_{a}=16.82\cdot A_{665.2}-9.28\cdot A_{652.4}$$

(2) $$C_{b}=36.92\cdot A_{646.8}-16.54\cdot A_{663.2}$$

where *C_a_* = chlorophyll *a* (μg/mL); *C_b_* = chlorophyll *b* (μg/mL); A_665.2_ = absorbance at 665.2 nm; A_652.4_ = absorbance 652.4 nm.

#### 3.2.3. Determination of Total Phenolic Compounds

The determination of total phenolic compounds (TPC) was carried out according to the colorimetric method with the Folin−Ciocalteu reagent, proposed by Singleton, Orthofer, and Lamuela-Raventos [60]. An aliquot of 500 μL of 7% ethanolic extract of the leaves was added to 1250 μL of 10% Folin–Ciocalteu solution (*v*/*v*), and after two minutes of reaction in the dark, 1000 μL of the 7.5% sodium carbonate solution (*w*/*v*) were added to the mixture. Proceeding 30 min of reaction at room temperature and protected from light, the solution was read in the spectrophotometer at 760 nm. To quantify the TPC, an analytical curve of Gallic acid was used, obtained in the concentration range from 20 to 100 mg/L. TPCs were expressed in mg Gallic acid equivalent per g of sample (mg GAE/g of sample).

#### 3.2.4. Determination of Total Flavonoids

The quantification of total flavonoids (TF) was carried out according to Pekal and Pyrzynska [61], with modifications. For this, 500 μL of 50% ethanolic extract of the leaves were added to 500 μL of 2% ethanolic aluminum chloride solution (*w*/*v*). After 10 min of reaction, at room temperature, and protected from light, the solution was read in a spectrophotometer at 425 nm. The analytical curve used to quantify TF was constructed in the concentration range from 5 to 200 mg/L, for rutin. Thus, the TF content was expressed in mg equivalent of rutin per gram of sample (mg ER/g of sample).

#### 3.2.5. Color Measurements

The color was analyzed by *tristimulus* colorimetry in a digital colorimeter (Choma Mater CR-300, Konica Minolta, Osaka, Japan), by the CIELAB coordinate system (L*, a*, b*), using the 10° viewing angle and D65 illuminant standard. The L* coordinate refers to changes in luminosity, with a range from 0 = black to 100 = white; the chromatic coordinate a* represents the green–red dimension (−*a* = green and +*a* = red); and the chromatic coordinate b* is associated with the blue–yellow dimension (−*b* = blue and +*b* = yellow). The readings were performed in triplicate and, with the obtained values, the chroma (C*) (Equation (3)), the Hue angle (*h°*) (Equations (4) and (5)), and the total color difference (ΔE) (Equation (6)) were calculated [62].

(3) $$C^{*\;}={\left[{\left(a^{*}\right)}^{2}+\left(b^{*}\right)2\right]}^{1/2}$$

(4) $$\;h{}^{\circ}=\arctan\left(\frac{b^{*}}{a^{*}}\right)\;\;\;\;\left(para+a^{*}e+b^{*},\;quadrante\;I\right)$$

(5) $$\;h{}^{\circ}=180+\;\arctan\left(\frac{b^{*}}{a^{*}}\right)\;\;\;\;\left(para-a^{*}e+b^{*},\;quadrante\;II\right)$$

(6) $$\Delta E={\left[\left(\Delta a^{*}\right)+\left(\Delta b^{*}\right)+\left(\Delta L^{*}\right)\right]}^{1/2}$$

where C* = chroma; *h°* = Hue angle; ΔE = total color difference; a* and b* = color coordinates; L* = luminosity.

### 3.3. Drying of the Leaves

The basil leaves were ground in a multiprocessor for 2 min and 10 g samples were spread on aluminum trays, with known mass and dimensions. The material was submitted to convective drying in a tray dryer (Quimis-Q316M5), at temperatures of 40 °C, 50 °C, 60 °C, and 70 °C, which were chosen according to the literature [47,51,63]. To monitor the process, the set (tray + sample) was weighed every 10 min, in the first 30 min; every 15 min, for an additional 45 min; and every 30 min until reaching constant weight (mass variation less than 1%). The dried leaves were ground in a knife mill, placed in hermetically closed glass jars, and stored at room temperature (≈25 °C). The dry matter of the sample was determined at 105 °C. The drying curves were obtained by correlating the moisture ratio (*MR*) (Equation (7)) with the drying time.

(7) $$MR=\frac{X-X_{e}}{X_{i}-X_{e}}$$

where *MR* = moisture ratio (dimensionless); X = moisture content at time t (g/100 g dry basis—db); X_i_ = initial moisture content (g/100 g db); X_e_ = equilibrium moisture content (g/100 g db).

In the dried leaves, analyses of chlorophyll *a*, chlorophyll *b*, total phenolic compounds, total flavonoids, and instrumental color were carried out according to the methodologies described in Section 3.2.

#### 3.3.1. Calculation of Effective Diffusivity and Activation Energy

The value of effective diffusivity (*D_eff_*), a property that characterizes the mass transfer during the drying of a material, was determined from Fick’s second law of diffusion (Equation (8)) [64]. It is an important parameter that indicates the transfer of water within the product, and varies with drying conditions, so it is not intrinsic to the material [65]. The calculation was performed by linear regression, considering the temperature of the drying air and a bed thickness of 4 mm. Additionally, the activation energy (*E_a_*) was calculated by the equation 9. The *E_a_* value indicates the degree of water diffusivity in the material during drying [64].

(8) $$MR=\frac{8}{\pi^{2}}\;\exp\left(-\frac{D_{eff}\;\pi^{2}}{L^{2}}\;t\right)$$

where *MR* = moisture ratio (dimensionless); *D_eff_* = effective diffusivity coefficient (m^2^/s); *L* = bed thickness (m); *t* = drying time (s).

(9) $$\;D_{eff\;}=D_{0\;\;}\exp\left(-\frac{E_{a}}{R\;T}\right)$$

where *D_eff_* = effective diffusivity (m^2^/s); *D_0_* = pre-exponential factor of the Arrhenius-type equation (m^2^/s); *E_a_* = activation energy (J/mol); *R* = Universal gas constant (J/mol K); *T* = absolute temperature of the air-drying (K).

#### 3.3.2. Mathematical Modeling of Drying

The experimental data on the drying of purple basil leaves were submitted to mathematical modeling. To this end, the mathematical fits of the semi-empirical models presented in Table 5 were evaluated. These models are used to describe the thin layer drying behavior [66].

The coefficient of determination (Equation (18)), the reduced chi-square value (Equation (19)), and the relative mean square error (Equation (20)) were used to analyze the quality of the models’ fits.

(18) $$R^{2}=\frac{\sum_{i=1}^{N}\left(Y_{i}-Y_{pre,i}\;\right).\sum_{i=1}^{N}\left(Y_{i}-Y_{exp,i}\;\right)}{\sqrt{\left[\sum_{i=1}^{N}{\left(Y_{i}-Y_{pre,i}\right)}^{2}\right]}.\left[\sum_{i=1}^{N}{\left(Y_{i}-Y_{exp,i}\right)}^{2}\right]}$$

(19) $$\chi^{2}=\frac{\sum_{i=1}^{N}{\left(Y_{exp,i}-Y_{pre,i}\right)}^{2}}{N-n}$$

(20) $$\mathrm{RMSE}={\left[\frac{1}{N}\;\overset{N}{\underset{i=1}{\sum }}{\left(Y_{exp,i}-Y_{pre,i}\right)}^{2}\right]}^{1/2}$$

where R^2^ = coefficient of determination; χ^2^ = reduced chi-square; RMSE = root mean square error; *Y*_*exp*_ = experimental values; *Y*_*pre*_ = values predicted by the model; *N* = number of observations; *n* = number of model parameters.

### 3.4. Hygroscopic Behavior of Dry Leaves

#### 3.4.1. Obtaining Moisture Sorption Isotherms

To evaluate the hygroscopic behavior of the dried purple basil leaves, moisture adsorption, and desorption isotherms were obtained at 25 °C. Moisture sorption data were obtained by the DVS method (Dynamic vapor sorption), in the vapor sorption analyzer (VSA) equipment (Aqualab VSA, Decagon, Puma, WA, USA). A sample of the crushed dried leaves was weighed (≈650 mg), in a stainless steel capsule, on the analytical microbalance of the equipment, to obtain moisture adsorption and desorption data, in a range from 0.1 to 0.9 a_W_. Equilibrium data were obtained at different levels of relative humidity (RH) induced by changes in injections of dry steam and saturated steam. The equilibrium condition was set when three successive measures were below 0.05% (dm/dt%), where dm/dt is the ratio between mass variation and time variation, in percentage, between successive measurements. At the end of the process, the dry matter of the sample was determined at 105 °C [67].

#### 3.4.2. Determination of Monolayer Moisture Content

The monolayer moisture content (*m_o_*) was determined by linear regression, from the linearized form of the BET equation (Equation (21)) [68].

(21) $$\frac{\;a_{w}}{\left(1-a_{w}\right)\cdot m}=\frac{1}{m_{o\;}\cdot c}+\frac{\left(c-1\right)}{m_{o\;}\cdot c}\cdot a_{w}$$

where a_w_ = water activity (dimensionless); *m* = equilibrium moisture content (g H_2_O/100 g db); *m_o_* = monolayer moisture content (g H_2_O/100 g db); *c* = constant related to the heat of sorption.

#### 3.4.3. Mathematical Modeling of Moisture Sorption Isotherms

The moisture adsorption and desorption data were submitted to mathematical modeling, in which the fits of the six models shown in Table 6, were evaluated. The coefficient of determination (Equation (18)), the mean squared relative error (Equation (20)) and the mean absolute percentage error (Equation (22)) were used to assess the quality of the fits.

(22) $$P=\frac{100}{N}\;\overset{N}{\underset{i=1}{\sum }}\frac{|Y_{exp}-Y_{pre}|}{Y_{exp}}$$

where P = mean absolute percent error; *Y_exp_* = experimental values; *Y_pre_* = values predicted by the model; *N* = number of observations.

### 3.5. Statistical Analysis

The results of the properties evaluated under different conditions were submitted to analysis of variance (ANOVA) and to Tukey’s test for the means comparison, at a confidence of 95%. The fits of the drying and moisture sorption models to the experimental data were performed by non-linear regression, using the Levenberg–Marquardt estimation methodology, with a convergence criterion of 10^−6^. All statistical analyses were performed using the Statistica 7.0 program.

## 4. Conclusions

According to the results, convective drying is suitable for reducing a_w_, securing degradative stability, and prolonging the shelf life of purple basil leaves. The *D_eff_* values indicated that the increase in temperature (from 40 °C to 70 °C) favors the elimination of water from the leaves, but it causes losses of chlorophyll and phenolic compounds. Drying at 40 °C promoted the lowest losses of TPC (26.1%) and a change in the color of the leaves, but drying in this temperature is a slow process, not suitable for practical purposes. For thermosensitive conservation purposes, drying at 40 °C is recommended. Modified Page, Diffusion Approximation, and Verna models were efficient in predicting the drying kinetics of purple basil leaves. The moisture adsorption isotherm showed that dried purple basil leaves are more susceptible to water gain in an environment with relative humidity above 50%, and that the dried leaves present microbiological stability in moisture content less than 8.83 g H_2_O/100 g db, if stored at 25 °C. In addition, the moisture desorption isotherm indicated that the purple basil leaves should not be dried to moisture contents lower than 5.96 g H_2_O/100 g db. The Oswin mathematical model is indicated to describe the moisture sorption isotherms of purple basil leaves. Finally, this study can contribute to future research that is required for the drying of purple basil leaves, for applications in food preparation, essential oil extraction, and preparation of infusions, among others, as well as for the storage of these leaves.

## Figures and Tables

**Table 1:** Proximate composition and physical–chemical properties of the purple basil leaf (*Ocimum basilicum* L.).

Properties *	Mean ± Standard Deviation
Moisture content (%)	83.56 ± 0.26
Ash (%)	1.63 ± 0.03
Total proteins (%)	0.57 ± 0.03
Total lipids (%)	0.15 ± <0.01
Total fibers (%)	10.97 ± 0.37
Total carbohydrates (%)	3.14 ± 0.27
Total energetic value (kcal/100 g)	16.11 ± 0.96
a_w_ at 25 °C	0.99 ± <0.01
pH	6.73 ± 0.06
Total titratable acidity (mEq NaOH/100 g)	0.72 ± 0.03
Total soluble solids (°Brix)	5.20 ± 0.40

* Values on a wet basis.

**Table 2:** Statistics modeling and parameters of the mathematical models adjusted to the drying data of the purple basil leaves.

Drying Temperature	Mathematical Model	Adjustment Parameters			
		Coefficients	R^2^	χ^2^	RMSE
40 °C	Newton	k = 0.0043	0.953	0.0063	0.08
	Modified Page	k = 0.0041 *n* = 1.54	0.990	0.0014	0.04
	Henderson and Pabis	a = 1.10 k = 0.0046	0.960	0.0056	0.07
	Two-term Exponential	a = 0.0016 k = 2.62	0.953	0.0067	0.08
	Logarithmic	a = 1.58 k = 0.0020 c = −0.5687	0.993	0.0010	0.03
	Diffusion approximation	a = 6.51 k = 0.0006 b = 0.7313	0.993	0.0010	0.03
	Verna	a = −1.77 k = 0.0006 g = 0.0015	0.993	0.0010	0.03
	Two-term	a = 0.5752 k_o_ = 0.0046 b = 0.4944 k = 0.0046	0.960	0.0062	0.07
50 °C	Newton	k = 0.0086	0.972	0.0042	0.06
	Modified Page	k = 0.0083 *n* = 1.38	0.993	0.0011	0.03
	Henderson and Pabis	a = 1.07 k = 0.0092	0.977	0.0036	0.06
	Two-term Exponential	a = 0.0009 k = 8.99	0.972	0.0045	0.06
	Logarithmic	a = 1.18 k = 0.0067 c = −0.1426	0.990	0.0016	0.04
	Diffusion approximation	a = −19.16 k = 0.0165 b = 0.9602	0.992	0.0013	0.03
	Verna	a = 11.49 k = 0.0156 g = 0.0168	0.992	0.0013	0.03
	Two-term	a = 0.8759 k_o_ = 0.0092 b = 0.1901 k = 0.0092	0.977	0.0041	0.06
60 °C	Newton	k = 0.0118	0.969	0.0048	0.07
	Modified Page	k = 0.0116 *n* = 1.45	0.996	0.0007	0.02
	Henderson and Pabis	a = 1.08 k = 0.0128	0.977	0.0039	0.06
	Two-term Exponential	a = 0.0012 k = 9.49	0.969	0.0052	0.07
	Logarithmic	a = 1.17 k = 0.0100 c = −0.1154	0.988	0.0022	0.04
	Diffusion approximation	a = −17.23 k = 0.0243 b = 0.9518	0.994	0.0010	0.03
	Verna	a = −7.36 k = 0.0251 g = 0.0225	0.994	0.0010	0.03
	Two-term	a = 0.2688 k_o_ = 0.0128 b = 0.8119 k = 0.0128	0.977	0.0047	0.06
70 °C	Newton	k = 0.0159	0.964	0.0057	0.07
	Modified Page	k = 0.0161 *n* = 1.56	0.998	0.0004	0.02
	Henderson and Pabis	a = 1.09 k = 0.0175	0.974	0.0046	0.06
	Two-term Exponential	a = 0.0012 k = 12.62	0.964	0.0063	0.07
	Logarithmic	a = 1.16 k = 0.0145 c = −0.0907	0.983	0.0033	0.05
	Diffusion approximation	a = −29.39 k = 0.0351 b = 0.9678	0.995	0.0009	0.03
	Verna	a = −10.23 k = 0.0362 g = 0.0330	0.995	0.0009	0.03
	Two-term	a = 0.2110 k_o_ = 0.0175 b = 0.8820 k = 0.0175	0.974	0.0057	0.06

**Table 3:** Instrumental color parameters for purple basil in natura and dried at different temperatures.

Samples (Leaves)	Instrumental Color Parameters					
	a*	b*	L*	C*	*h°*	ΔE
In natura	−13.99 ^a^ ± 0.66	21.33 ^a^ ± 0.02	34.20 ^a^ ± 1.27	25.52 ^a^ ± 1.96	123.33 ^a^ ± 1.89	−
Dried at 40 °C	0.24 ^b^ ± 0.04	12.35 ^b^ ± 0.53	26.55 ^b^ ± 0.85	12.35 ^b^ ± 0.53	88.61 ^b^ ± 1.09	1.78 ^c^ ± 0.08
Dried at 50 °C	0.29 ^b^ ± 0.08	10.99 ^bc^ ± 0.45	23.50 ^c^ ± 0.76	10.99 ^bc^ ± 0.45	88.46 ^b^ ± 0.47	2.59 ^b^ ± 0.21
Dried at 60 °C	0.07 ^b^ ± 0.01	11.33 ^b^ ± 0.13	23.68 ^c^ ± 0.97	11.33 ^b^ ± 0.13	89.97 ^b^ ± 0.42	2.53 ^b^ ± 0.21
Dried at 70 °C	0.27 ^b^ ± 0.04	8.63 ^c^ ± 0.19	23.01 ^c^ ± 0.77	8.63 ^c^ ± 0.19	88.19 ^b^ ± 0.28	3.10 ^a^ ± 0.14

Means with the same letters in the same column do not differ statistically (*p* ≤ 0.05) for the Tukey’s test.

**Table 4:** Statistics and model’s coefficients for mathematical modeling of moisture sorption data of dried purple basil leaves at 25 °C.

Isotherm	Mathematical Model	Adjustment Parameters			
		Coefficient	R^2^	P (%)	RMSE
Adsorption	Halsey	a = 12.01 b = 1.47	0.996	6.38	0.339
	Henderson	a = 7.23 b = 0.54	0.982	11.15	0.715
	Oswin	a = 7.23 b = 0.54	0.998	2.72	0.213
	Smith	a = 1.30 b = 8.79	0.991	4.81	0.501
	GAB	m_o_ = 4.29 c = 10.45 k = 0.91	0.999	2.35	0.213
	Peleg	a = 28.25 b = 12.15 c = 7.88 d = 0.76	0.999	2.69	0.135
Desorption	Halsey	a = 77.77 b = 2.08	0.980	8.19	0.684
	Henderson	a = 0.01 b = 1.77	0.993	3.55	0.396
	Oswin	a = 10.08 b = 0.37	0.997	3.09	0.253
	Smith	a = 4.26 b = 7.94	0.989	6.35	0.510
	GAB	m_o_ = 7.05 c = 14.08 k = 0.76	0.998	2.15	0.228
	Peleg	a = 15.15 b = 15.30 c = 6.64 d = 0.59	0.999	1.70	0.147

**Table 5:** Kinetic models used to describe the drying curves of purple basil leaves.

Model	Equation		Number of Parameters
Newton	$MR=e^{-\mathrm{kt}}$	(10)	1
Modified Page	$MR=e^{-{\left(\mathrm{kt}\right)}^{n}}$	(11)	2
Henderson and Pabis	$MR={a\;e}^{-\mathrm{kt}}$	(12)	2
Two-term exponential	$MR={a\;e}^{-\mathrm{kt}}+\left(1-a\right){\;e}^{-\mathrm{akt}}$	(13)	2
Logarithmic	$MR={a\;e}^{-\mathrm{kt}}+c$	(14)	3
Diffusion approximation	$MR={a\;e}^{-\mathrm{kt}}+\left(1-a\right){\;e}^{-\mathrm{bkt}}$	(15)	3
Verna	$MR={a\;e}^{-\mathrm{kt}}+\left(1-a\right){\;e}^{-\mathrm{gt}}$	(16)	3
Two-term	$MR={a\;e}^{-k_{0}t}+{b\;e}^{-k_{1}t}$	(17)	4

Source: Inyang et al. [66].

**Table 6:** Mathematical models used to describe the moisture sorption isotherms of purple basil leaves.

Model	Equation		Number of Parameters
Halsey *	$m={\left[\frac{-a}{{\mathrm{lna}}_{w}}\right]}^{\frac{1}{b}}$	(23)	2
Henderson *	$m={\left[\frac{-\ln\left(1-a_{w}\right)}{{\mathrm{lna}}_{w}}\right]}^{\frac{1}{b}}$	(24)	2
Oswin *	$m=a{\left[\frac{a_{w}}{1-a_{w}}\right]}^{b}$	(25)	2
Smith *	$m=a-b\cdot \ln\left(1-a_{w}\right)$	(26)	2
GAB **	$m=\frac{m_{o}\cdot c\cdot a_{w}}{\left[\left(1-k\cdot a_{w}\right)\cdot \left(1+\left(c-1\right)\cdot k\cdot a_{w}\right)\right]}$	(27)	3
Peleg ***	$m=a\cdot a_{w}^{c}+b\cdot a_{w}^{d}$	(28)	4

* Chirife and Iglesias [69]; ** Maroulis et al. [70]; *** Peleg [71].

## Data Availability

Not applicable.
